# Plantar Pressure in Diabetic Peripheral Neuropathy Patients with Active Foot Ulceration, Previous Ulceration and No History of Ulceration: A Meta-Analysis of Observational Studies

**DOI:** 10.1371/journal.pone.0099050

**Published:** 2014-06-10

**Authors:** Malindu Eranga Fernando, Robert George Crowther, Elise Pappas, Peter Anthony Lazzarini, Margaret Cunningham, Kunwarjit Singh Sangla, Petra Buttner, Jonathan Golledge

**Affiliations:** 1 Vascular Biology Unit, Queensland Research Centre for Peripheral Vascular Disease, School of Medicine and Dentistry, James Cook University, Townsville, Queensland, Australia; 2 Movement Analysis Laboratory, Institute of Sports and Exercise Science, James Cook University, Townsville, Queensland, Australia; 3 Department of Internal Medicine, The Townsville Hospital, Townsville, Queensland, Australia; 4 Allied Health Research Collaborative, Metro North Hospital & Health Service, Queensland Health, Brisbane, Queensland, Australia; 5 School of Clinical Sciences, Queensland University of Technology, Brisbane, Queensland, Australia; 6 School of Public Health and Tropical Medicine, James Cook University, Townsville, Queensland, Australia; 7 Department of Vascular and Endovascular Surgery, The Townsville Hospital, Townsville, Queensland, Australia; 8 Department of Psychology, University of Stirling, Stirling, Scotland, United Kingdom; Sapienza, University of Rome, School of Medicine and Psycology, Italy; di Pompeo d'Illasi

## Abstract

**Aims:**

Elevated dynamic plantar pressures are a consistent finding in diabetes patients with peripheral neuropathy with implications for plantar foot ulceration. This meta-analysis aimed to compare the plantar pressures of diabetes patients that had peripheral neuropathy and those with neuropathy with active or previous foot ulcers.

**Methods:**

Published articles were identified from Medline via OVID, CINAHL, SCOPUS, INFORMIT, Cochrane Central EMBASE via OVID and Web of Science via ISI Web of Knowledge bibliographic databases. Observational studies reporting barefoot dynamic plantar pressure in adults with diabetic peripheral neuropathy, where at least one group had a history of plantar foot ulcers were included. Interventional studies, shod plantar pressure studies and studies not published in English were excluded. Overall mean peak plantar pressure (MPP) and pressure time integral (PTI) were primary outcomes. The six secondary outcomes were MPP and PTI at the rear foot, mid foot and fore foot. The protocol of the meta-analysis was published with PROPSERO, (registration number CRD42013004310).

**Results:**

Eight observational studies were included. Overall MPP and PTI were greater in diabetic peripheral neuropathy patients with foot ulceration compared to those without ulceration (standardised mean difference 0.551, 95% CI 0.290–0.811, p<0.001; and 0.762, 95% CI 0.303–1.221, p = 0.001, respectively). Sub-group analyses demonstrated no significant difference in MPP for those with neuropathy with active ulceration compared to those without ulcers. A significant difference in MPP was found for those with neuropathy with a past history of ulceration compared to those without ulcers; (0.467, 95% CI 0.181– 0.753, p = 0.001). Statistical heterogeneity between studies was moderate.

**Conclusions:**

Plantar pressures appear to be significantly higher in patients with diabetic peripheral neuropathy with a history of foot ulceration compared to those with diabetic neuropathy without a history of ulceration. More homogenous data is needed to confirm these findings.

## Introduction

One of the most detrimental complications of both Type 1 and Type 2 diabetes mellitus (DM) is foot ulceration [1], [2]. The prevalence of diabetes foot ulceration (DFU) in the United States ranges between 4 and 12%, the annual incidence ranges between 1 and 4.1% and the lifetime incidence can be as high as 25% [3]. DFU is caused by the interplay of several factors, but most notably diabetic peripheral neuropathy (DPN), peripheral arterial disease (PAD) and changes in foot structure, resulting in foot deformity and increased weight bearing pressure [4]–[6].

DFU proceeds up to 85% of all amputations in DM patients and the estimated likelihood of lower limb amputation is 10 to 30 times higher amongst patients with DM, compared to non-DM counterparts [3], [8]. Substantial healthcare costs are associated with foot ulcer treatment and prevention in both developed and developing countries and this is a global concern [7], [9]. Foot ulceration together with underlying disease processes such as cardiovascular disease impacts on health related quality of life and ambulatory status in people with DM, promoting decline in general health, mental health, physical and social functioning [7], [10], . Therefore, greater understanding of the factors precipitating DFU is urgently needed.

Raised dynamic plantar pressures are a frequent finding in DM patients with neuropathy [12]–[14]. Two measures of vertical plantar pressure are most commonly assessed. Mean peak plantar pressure (MPP) represents the maximum amount of pressure during stance and the mean pressure time integral (PTI) represents the amount of time over which maximum pressure is applied [15]. A recent meta-analysis of observational studies by our team demonstrated significantly higher PTI and MPP in DPN patients when compared to healthy and DM controls that did not have neuropathy [16].

There has been extensive interest regarding the role of plantar pressures and pressure offloading in foot ulceration and the ability to determine a plantar pressure cut-off which predicts ulceration [17]–[26]. Several observational studies have investigated the feasibility of using plantar pressure in identifying those at risk of ulceration, but the reported sensitivities and specificities are below those which are typically accepted for a diagnostic test [23], [24], [25]. Nevertheless, if patients with warning signs of impending foot ulceration could be identified using plantar pressures, alongside other confirmed risk factors; it is possible that clinical management could be improved to avoid foot ulcer development.

Prior to assessing plantar pressure as a screening tool for ulceration, it would appear necessary to determine whether plantar pressures are actually significantly higher in patients with DPN with previous and/or present diabetes foot ulceration (PPDFU) compared to patients with DPN without a history of DFU. The relatively low specificity and sensitivity values obtained in the aforementioned studies raises the question as to whether there is an increase in plantar pressure prior to the onset of ulceration and following ulceration in those with DPN. To the best of the authors' knowledge a meta-analysis to examine these questions has not been previously published.

The primary aim of this meta-analysis was to compare plantar pressures in patients with PPDFU (cases) and individuals with DPN without a history of ulceration (controls). The secondary aims were to assess the quality of studies investigating plantar pressure and to investigate plantar pressure in patients with active and past ulcers.

## Methods

### Search strategy and quality assessment

A comprehensive search strategy was utilised, involving MeSH and Emtree terms and relevant keywords for search strings (see Figure S1). The databases searched were Medline via OVID (1946 to present), CINAHL (1994–2012), SCOPUS (all years to present), INFORMIT, Cochrane Central (latest), EMBASE via OVID (1980 to present) and Web of Science via ISI Web of Knowledge (1965 to present). One author (MF) and a librarian carried out the searches (using the same search string) independently on two separate occasions in November 2012 to identify all relevant studies published until 18th November 2012. Furthermore, a repeated search was conducted in February 2014 to identify recent studies of interest for inclusion. No new articles of relevance were found after the latter search.

It has been identified that a single candidate tool for the quality assessment of observational studies does not exist [27]. Assessment of risk of bias was conducted using a quality assessment tool adapted from validated instruments (Pedro and CASP) with the addition of content specific questions concerning plantar pressure and foot ulceration [28]–[30]. Risk of bias was assessed by two blinded authors (PL and EP) who were given study manuscripts after the removal of author, institutional, title and re-identifiable information. The quality scores were then checked by one author (MF) for consistency. Where major differences in quality scores existed, these were discussed amongst a second group of authors (MF, MC and PB) and the original blinded quality assessors were asked to independently review any major differences in ratings. The quality assessment tool was trialled by the two assessors prior to use in the meta-analysis. An abbreviated version of the quality assessment tool used is shown in Table S1 in File S1. As an adapted quality assessment tool was used a total score of 50 was possible for the 25 questions. Quality scores of ≥45, 30–45, 20–30 and ≤20 were defined as excellent, good, fair and poor respectively.

### Study selection

Studies were included in the meta-analysis if they met all the below inclusion criteria:

- An observational study;
- Subjects included were adults aged 18 years and over;
- The study was reported in or available in the English language;
- The study used a validated method of diagnosing DPN, including one or more of the following methods; A screening questionnaire to assess DPN; 10 g monofilament perception testing; vibration perception threshold; and nerve conduction studies;
- Plantar pressures were reported in two groups of subjects with documented DPN in which at least one group had a previous or active plantar neuropathic foot ulcer;
- Barefoot dynamic plantar pressure during walking was reported without the influence of an offloading intervention or footwear.
- Plantar pressure values were reported as either MPP and/or PTI in any acceptable pressure unit (KPa, N/kg^2^ or similar);
- Overall, fore foot, mid foot and/or rear foot MPP or PTI were reported; Studies were excluded if meeting any of the below criteria:
- The study was an interventional study. The authors considered interventional studies utilising offloading options (such as foot wear, insoles and orthoses) and or/treatment options (such as podiatry treatment, debridement of callus) as well as the assessment of plantar pressure could potentially alter the natural gait cycle and plantar pressures, and thus were excluded;
- The study did not document DPN status or where all the participants in a single group did not have documented DPN;
- Data could not be extracted for the two groups of interest or computed, or where authors were unable to provide data for the two groups of interest from a larger dataset when requested;
- Only in shoe plantar pressure were reported, as these were considered interventional assessments and the combination of shod and barefoot findings can drastically increase the variability of plantar pressure results [26];
- Full text manuscripts could not be acquired.

The two primary outcomes of this study were overall MPP and overall mean PTI. The six secondary outcomes were MPP at the rear foot, MPP at the mid foot, MPP at the fore foot, PTI at the rear foot, PTI at the mid foot and PTI at the fore foot.

Potential studies identified for inclusion were reviewed independently by 3 authors (MF, RC and MC) using the above inclusion criteria. Where there was disagreement in the inclusion of studies, group discussions were held to resolve any differences in opinion. All reference lists of studies meeting the inclusion criteria were browsed for any additional studies for inclusion. Authors of the included studies were likewise contacted via email for identification of other potential studies for inclusion. No further studies of relevance were found.

### Data extraction and synthesis

Data extraction was completed by the primary author, using specifically developed data extraction forms. The forms were then checked by two authors (EP and PL) for any omissions. Descriptive data, such as age, gender, body mass index (BMI), and disease specific data, such as DM duration, type of DM, HbA1c, degree of neuropathy, presence of PAD, and presence of foot deformity were extracted from each study for comparison. Numerical data (mean and standard deviation [SD]) for each plantar pressure variable (MPP and/or PTI) were also extracted and included in the analysis. Where anatomical locations were unspecified, or when overall MPP was the only variable reported, authors were contacted for plantar pressure data specific to different plantar locations.

The MOOSE reporting guidelines for meta-analysis of observational studies was used in the synthesis of this manuscript [31]. The protocol of the meta-analysis was registered and published with PROSPERO database of systematic reviews and meta-analyses prior to completing data extraction (registration number CRD42013004310). Meta-analysis was attempted where more than three studies reported a given outcome measure. Random effects models were used to analyse the studies based on the prospect there would be between study heterogeneity present.

### Statistical methods

Standardised mean differences were used in the computation of meta-analyses of plantar pressure differences utilising Cohen's d [32]. Results were reported as standardised mean differences with 95% confidence intervals (95%-CI) and p-values. Where SD was not reported, standard error (SE), inter-quartile range (IQR) or equivalent was converted to SD values. Since both Boulton et al. and Cavanagh and colleagues [33], [34] did not report SD for the distribution of plantar pressure and were unable to provide information about SD when contacted, these were estimated using linear regression of SD on mean values for MPP. However as the highest reported aggregate plantar pressure was 83.1 N/cm^2^, these estimations are approximations only and sensitivity analyses were conducted excluding these studies. As Stess et al. reported SE [35], SD was calculated using the formula SE = SD/√n. Similarly, as Sauseng et al. reported IQR [36], SD was estimated using the IQR value and PASS statistical software (NCSS LLC; Kaysville Utah).

Weighted means (according to the sample size of the studies) were calculated for the reported demographic variables. A Cohen's d score of zero was interpreted as no difference in effect; a result of 0 to 0.2 was interpreted as a small effect, 0.2–0.8 as a moderate effect and ≥0.8 as a large effect [37]. All statistical analyses were carried out by an experienced statistician (PB) using the software package Comprehensive Meta-Analysis (www.Meta-Analysis.com, USA).

In order to obtain any missing data or to clarify any discrepancies in data, several attempts were made to contact the corresponding authors by email, using open-ended questions. Where multiple studies were published from the same data set, the study with the most information reported in relation to the outcome measures of interest was used. Where more than one publication reported data from a single study, the publication with the largest data set was used. The primary unit of analysis for the meta-analysis was the patient. Where the unit of analysis were feet instead of patients, caution was taken in the interpretation of the results and authors were contacted for clarification. Rich et al. reported findings as number of feet instead of number of patients and the authors were unable to provide information specific to the patients [38].

The Q and I^2^ statistics were used to assess statistical heterogeneity between studies. I^2^ values of 25%, 50% and 75% were acknowledged as low, moderate and high heterogeneity respectively. In addition to this, the classic fail safe (N) was also computed; as this gives an estimation of studies needed to be published with a null effect to renounce the findings from the meta-analysis [39]. Sub-group analyses and sensitivity analyses were performed to examine the effect of including and excluding several studies. Sensitivity and sub-group analyses comprised of: Analyses including only active foot ulcer patients from the PPDFU group; analyses including only previous foot ulcer patients from the PPDFU group; analyses excluding the two studies for which SD was estimated [33], [34]; analyses excluding Rich et al. due to the difference in unit of analysis [38]; analyses with exclusion of Stess et al. due to the high level of statistical heterogeneity and the inclusion of amputees in the study [35]; and separate analyses with the exclusion of all three studies listed above.

## Results

### Search Results

**Figure 1:**
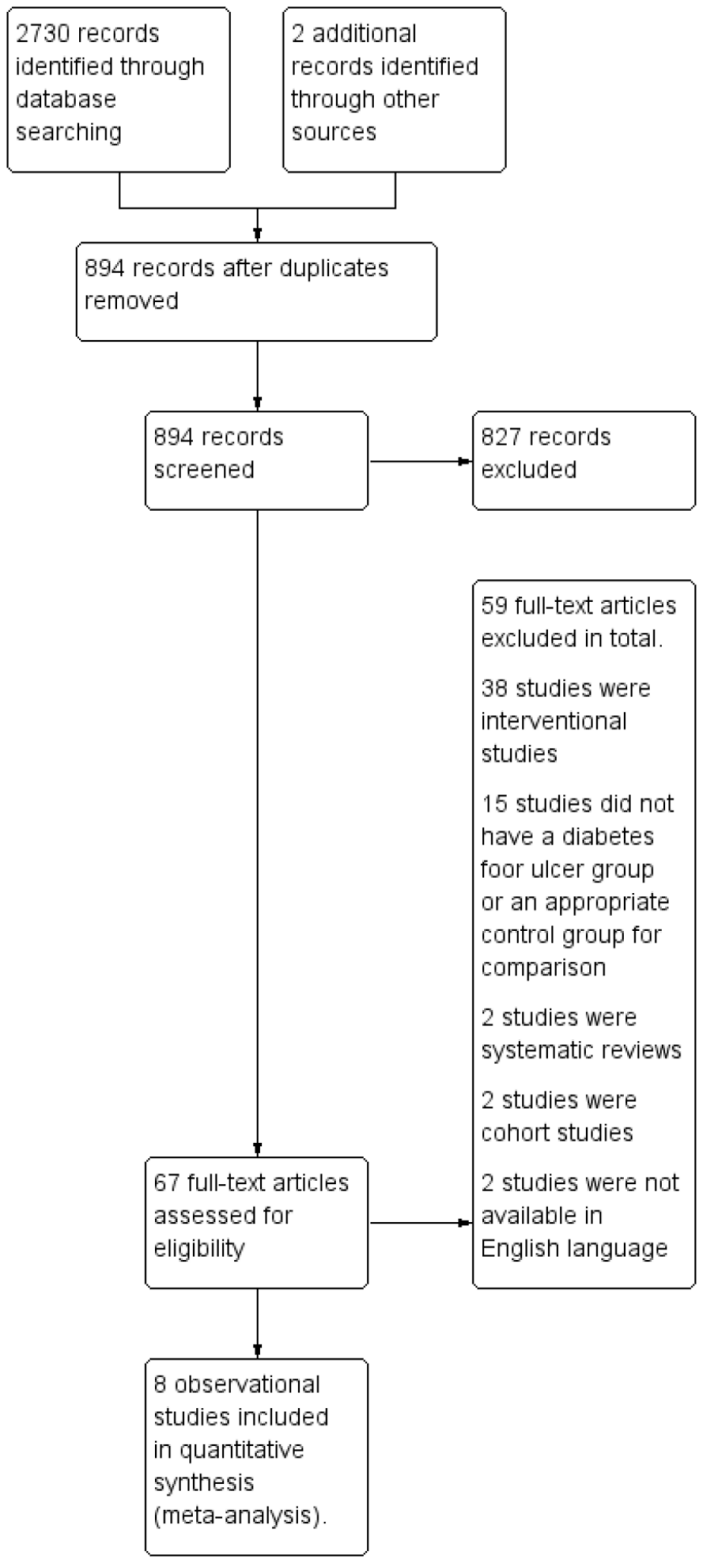
Search Results. Search results indicating total number of identified records (2730) and the number of articles remaining after duplicate removal (894) and the number of records excluded (827) and the number of full text articles assessed for eligibility according to the inclusion and exclusion criteria listed. This resulted in eight observational studies which were included in the meta-analysis.

The systematic search strategy resulted in the identification of 2730 citations. Figure 1 demonstrates a flow diagram representing the inclusion and exclusion of studies. The search strategy identified six studies for inclusion in the meta-analysis. A further two studies were identified and included after browsing through the reference lists of the included articles, resulting in a total of eight observational studies [23], [33]–[36], [38], [40]–[41].

### Description of studies

A comprehensive list of study characteristics can be found in Table 1. In total there were 647 DM participants from all eight studies. This included 238 PPDFU patients. The mean sample size of the PPDFU groups was 29.7 and ranged from 9 to 70 patients. The age range of the PPDFU patients was 52.3 to 62.4 years with a weighted mean age of 56.8 years. The majority of patients (77.3%) were men. The BMI of this group ranged from 27 Kg/m^2^ to 30.9 Kg/m^2^ (weighted mean 30.2 Kg/m^2^). The weighted mean duration of DM was 16.4 years with a range of 14.3 to 22.7 years. Sixty six percent of patients had active foot ulcers at the time of data collection and the remaining 34% were patients with a history of foot ulcers. The time period for which ulceration occurred varied between studies and most studies failed to report the time period which had elapsed since the last occurrence of ulceration in those with a history of ulcers. The monofilament perception threshold (MPT) was only reported by one study and was 2.89 [34]. The mean vibration perception threshold (VPT) in the PPDFU group was 37.6 V with a range of 33.5 to 40 V. Mean HbA1c was only reported by one study and was 8.8% or 73 mmol/mol [36]. Ankle brachial pressure index (ABPI) was reported by three studies and the weighted mean ABPI was 1.06 with a range of 0.96 to 1.44 [23], [33], [35].

**Table 1:** Characteristics of participants in included studies.

Study and (N)			Cases (PPDFU)										Controls (DPN)								
Study	N	DM Type	PPDFU Cases (n = )	Age (Yr.)	% Men	BMI (Kg/m^2^)	Diabetes Duration (years)	% Active Ulcer	MPT	VPT (V)	HbA1c (%)	ABPI	DPN Control (n = )	Age	% Men	BMI (Kg/m^2^)	Diabetes Duration (years)	MPT	VPT	HbA1c (%)	ABPI
**Bacarin 2009 ** [40]	**27**	T1, T2	**10**	58.2 (6.7)	80	27 (5.5)	17.5 (9.3)	0	-	-	-	-	**17**	54.7 (7.8)	47	26.1 (4.6)	13.4 (8.2)	-	-	-	-
**Cavanagh 1991 ** [34]	**56**	T1, T2	**14**	62.4	100	-	16.9 (9.2)^a^	100	2.89	39.3	-	-	**42**	57.8	100	-	16.9 (9.2)^a^	1.98	28.0	-	-
**Sauseng 1999 ** [36]	**43**	T1, T2	**20**	57.2	-	28.9 (4.3)	19.4 (8.5)	100	-	40 (14.0)	8.8 (1.8)		**23**	65.8 (7.0)	-	28.9 (4.6)	14.0 (7.5)	-	44.0(6.0)	8.0 (1.2)	
**Armstrong 1998 ** [23]	**219**	-	**70**	52.3 (10.3)	74	30.9 (5.7)	14.3 (9.2)	-	-	-	-	0.96 (0.17)	**149**	51.8 (10.4)	33	32.3 (6.2)	9.2 (8.8)	-	-	-	0.99 (0.16)
**Boulton 1983 ** [33]	**41**	-	**13**	57.4 (39.0)^a^	76	-	15.2 (15.0)^a^	0	-	35 (12.0)	-	1.44 (0.41)	**28**	49.9^a^ (18.1)	65	-	11.9 (21.1)^a^	-	30.0 (11.0)	-	1.30 (0.27)
**Rich 2000** b [38]	**180**	-	**53**	-	-	-	-	-	-	-	-		**127**	-	-	-	-	-	-	-	
**Brash 1996** d [41]	**18**	-	**9**	52.7(12.3)	-	-	22.7 (11.1)	100	-	33.5 (4.2)	-	-	**9**	56.5 (9.6)	-	-	22.1 (11.5)	-	31.0 (6.8)	-	-
**Stess 1997** c [35]	**63**	Majority T2	**49**	61.7 (12.4)	-	30.4 (2.7)	16.8	67	-	-	-	1.1 (0.3)	**14**	66.0 (8.9)	-	26.2 (1.1)	15.1	-	-	-	1.1 (0.2)
**Total & Weighted averages**			**238**	56.8; 52.3 – 62.4	77.3; 67–100	30.2; 27.0–30.9	16.4; 14.3–22.7	66; 0–100	2.89	37.6; 33.5–40.0	8.8	1.06; 0.96–1.44	**409**	54.7; 49.9–66.0	49.7; 33–100	31.0; 26.1–32.3	12.0; 9.2–22.1	1.98	32.4; 28.0–44.0	8.0	1.04; 0.99–1.30

Legend: Mean and (standard deviation SD) for PPDFU- Past present plantar diabetic foot ulcer (case), DPN- Diabetic peripheral neuropathy (control) groups. Weighted means (weighted by sample size) and ranges provided in final row.

MPT is the monofilament perception threshold and VPT is the vibration perception threshold (both used in the diagnosis of DPN), ABPI represents ankle brachial pressure index values. DM Type is diabetes mellitus type (type 1 = T1 and type 2 = T2).

a These variables were not described with SD but reported ranges, the difference between the maximum and mean (maximum minus the mean) are reported instead of SD.

b This study reported findings as number of feet instead of patients.

c 26% of PPDFU group had digital or metatarsal amputations. BMI (SD) was calculated from data provided in non-metric format.

d This study used an active ulcer group (33%) for PPDFU but utilised the non-ulcerated foot for measurement of plantar pressure.

The group of DPN patients without a history of foot ulcers consisted of 409 subjects. The mean sample size was 51.1 with a range of 9 to 149 patients. Overall the weighted mean age of this group was 54.7 years with a range of 49.9 to 66 years. The minority of subjects (49.7%) were men and the BMI range was 26.1 to 32.3 Kg/m^2^ (weighted mean 31.0 Kg/m^2^). The weighted mean DM duration was 12.0 years with a range of 9.2 to 22.1 years. The mean MPT value reported in one study was 1.98 [34]. The weighted mean VPT value was 32.4 V with a range of 28 to 44 V. The mean HbA1c, reported by one study was 8.0%, or 64 mmol/mol [36]. The mean ABPI of this group was 1.04 with a range of 0.99 to 1.30.

The method of diagnosing DPN varied in different studies. One study used monofilament testing alone at six plantar locations to identify DPN [35]. One study used neuropathic symptoms and an absent ankle reflex for the diagnoses of DPN [33], however, this was an acceptable means of diagnosing DPN at the time of the study. A few studies utilised neuropathy questionnaire(s) for the assessments of DPN, in addition to the VPT [38], [40], [41]. Only one study commented on the presence/absence of plantar callus on the feet of participants. Brash et al. identified the locations of plantar callus and found no significant difference in callus between the two groups studied [41].

### Risk of bias in included studies

The overall agreement between the two quality assessors was good; with the range of variation of scores between zero to three points (Table S1 in File S1). In general, all studies used an appropriate study design, accounted for potential confounders and reported data for at least 85% of the participants for a primary outcome measure. However, four studies failed to discuss the main sources of bias within the study [33], [35], [38], [41] and two studies did not identify the presence of PAD or exclude those with PAD [34], [40]. There was not an overall noteworthy difference in the methodological quality of studies. The highest score for the method and participant specific questions were given to two studies which addressed issues such as number of steps used in measurements, number of walking trials and the measurement of factors which potentially affected plantar pressure, such as foot structure [38], [40].

### Primary outcome measures

#### Overall MPP

**Figure 2:**
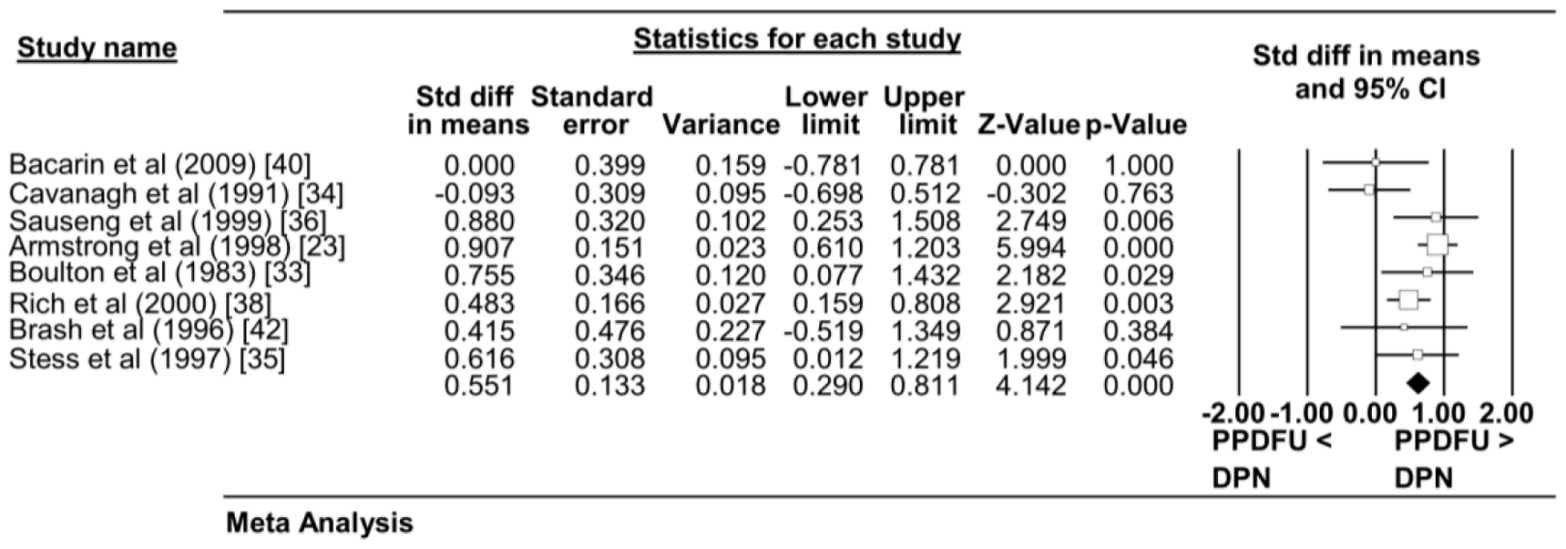
Forest Plot. Forest Plot displaying the Overall Peak Plantar Pressure (MPP) between the PPDFU group (cases) and the DPN group (control). Overall effect is represented by the coloured diagonal. Eight studies are included in total.

Overall MPP was reported by all eight studies. Table S2 in File S1 illustrates the reported plantar pressures according to plantar anatomical locations as well as the overall plantar pressures from each study. Meta-analysis combining data from eight studies (PPDFU n = 238; DPN with no foot ulcer history n = 409) suggested that patients with PPDFU had greater overall MPP with moderate effect levels (standardised mean difference 0.551, 95% CI 0.290–0.811; p≤0.001). The heterogeneity between studies was moderate I^2^ = 46.2 (Figure 2).

#### Overall PTI

**Figure 3:**
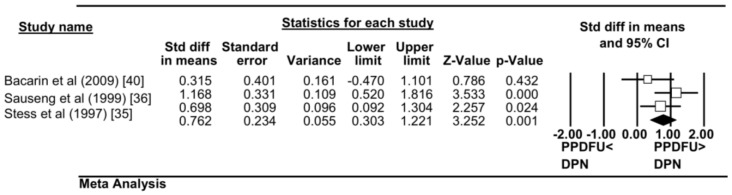
Forest Plot. Forest Plot displaying the Overall Pressure Time Integral (PTI) between the PPDFU group(cases) and the DPN group(control). Overall effect is represented by the coloured diagonal. Three studies are included in total.

Three studies reported PTI values (Table S2 in File S1). Meta-analysis combining data from all three studies (PPDFU n = 79; DPN with no foot ulcer history n = 54) suggested that patients with PPDFU had greater overall PTI with moderate effect levels (standardised mean difference 0.762, 95% CI 0.303–1.221; p = 0.001). The heterogeneity between studies was moderate I^2^ = 28.4 (Figure 3). As only three studies were found, sub-group and sensitivity-analyses were not attempted.

### Sensitivity and sub-group analyses

Meta-analysis combining data from three studies which reported plantar pressure in active ulcer patients (PPDFU [present ulcer only] n = 43; DPN with no foot ulcer history n = 74) suggested a non-significant difference for plantar pressure in patients with active foot ulcers compared to DPN participants without foot ulcers (Table 2). Furthermore, an analysis of three studies using patients with a history of foot ulcers [previous ulceration only] suggested that overall MPP was significantly higher in those with a history of ulcers (n = 76) versus DPN without a history of ulcers (n = 172), (standardised mean difference 0.467, 95% CI 0.181–0.753; p = 0.001) with low heterogeneity I^2^ = 3.6.

**Table 2:** Meta analyses results.

Outcome measure	Comparison	Number of studies	Effect size (95%-CI)	p-value	Heterogeneity assessment	Classic fail safe N
Overall Peak Plantar Pressure MPP [N/cm^2^]	PPDFU (n = 238) versus DPN (n = 409)	8^d^	0.551 (0.290, 0.811)	p<0.001	Q = 13.0; p = 0.072; I^2^ = 46.2	63
	PPDFU (n = 211) versus DPN (n = 339)	6d2	0.635 (0.387, 0.884)	p<0.001	Q = 7.3; p = 0.200; I^2^ = 31.4	49
	PPDFU (n = 185) versus DPN (n = 282)	7^a^	0.553 (0.229, 0.876)	p = 0.001	Q = 12.2; p = 0.058; I^2^ = 50.76	41
	PPDFU (n = 43) versus DPN (n = 74)	3^c^	0.394 (-0.237, 1.026)	p = 0.221	Q = 4.8; p = 0.091; I^2^ = 58.3	/
	PPDFU (n = 189) versus DPN (n = 395)	7^e^	0.534 (0.235, 0.832)	p<0.001	Q = 13.0; p = 0.043; I^2^ = 53.9	48
	PPDFU (n = 136) versus DPN (n = 268);	6f1	0.528 (0.143, 0.914)	p = 0.007	Q = 12.1; p = 0.033; I^2^ = 58.9	29
	PPDFU (n = 76) versus DPN (n = 172)	3^g^	0.467 (0.181, 0.753)	p = 0.001	Q = 2.1; P = 0.354; I2 = 3.6	4
Overall Peak Plantar Pressure PTI [Ns/cm^2^]	PPDFU (n = 79) versus DPN (n = 54)	3^b^	0.762 (0.303, 1.221)	p = 0.001	Q = 2.8; p = 0.248; I^2^ = 28.4	9
Forefoot MPP [N/cm^2^]	PPDFU (n = 211) versus DPN (n = 339)	6	0.635 (0.387, 0.884)	p<0.001	Q = 7.3; p = 0.200; I^2^ = 31.4	49
	PPDFU (n = 158) versus DPN (n = 212)	5^a^	0.692 (0.392, 0.992)	p<0.001	Q = 5.5; p = 0.243; I^2^ = 26.7	31
	PPDFU (n = 162) versus DPN (n = 325)	5^e^	0.625 (0.323, 0.927)	p<0.001	Q = 7.3; p = 0.123; I^2^ = 44.9	36
	PPDFU (n = 109) versus DPN (n = 198)	4f2	0.670 (0.273, 1.066)	p = 0.001	Q = 5.2; p = 0.157; I^2^ = 42.5	21
Forefoot PTI [Ns/cm^2^]	PPDFU (n = 79) versus DPN (n = 54)	3^b^	0.719 (0.197, 1.242)	p = 0.007	Q = 3.6; p = 0.165; I^2^ = 44.4	8

Legend: Random effects model meta-analyses. Effect size is standardised difference of mean values calculated as (DPN – PPDFU). Hence a negative result implies smaller values for DPN.

a Rich et al. (2000) excluded because of issues with unit of analysis;

b Studies included Bacarin, Sauseng and Stess;

c Analysis of studies with 100% active ulcer group [active ulcer only] (Cavanagh et al Sauseng et al and Brash et al);

d All studies (n = 8) including Cavanagh and Boulton with SD estimated from linear regression;

d2 Excluding Cavanagh and Boulton with SD estimated from linear regression;

e Stess et al (1997) excluded due to high level of heterogeneity and inclusion of amputees;

f1 Rich et al and Stess et al excluded due to reasons given above;

f2 Rich et al and Stess et al excluded due to reasons given above;

g Analysis with history of ulcers only [excluding active ulceration] (Bacarin et al, Boulton et al and Rich et al).

A sensitivity analysis was performed for overall MPP excluding studies by Cavanagh et al. and Boulton et al. [33], [34] as their SD was estimated using linear regression (Table S2 in File S1). This resulted in a small increase in effect size (standardised mean difference 0.635, 95% CI 0.387–0.884; p≤0.001), and reduction in heterogeneity I^2^ = 31.4. Removal of the study by Rich et al. [38] (Table 2) due to unit of analysis difference resulted in a small increase in effect size but an increase in heterogeneity to I^2^ = 50.76. Exclusion of the study by Stess et al. [35] (due to the inclusion of foot amputees in the PPDFU group) created a minute reduction in effect size and an increase in heterogeneity levels (I^2^ = 53.9) (Table 2). The exclusion of two major studies potentially causing heterogeneity (Stess et al. and Rich et al. [35], [38]) resulted in a small reduction in effect size and a substantial increase in heterogeneity I^2^ = 58.9 (Table 2).

### Secondary outcome measures

#### MPP at various plantar foot regions

**Figure 4:**
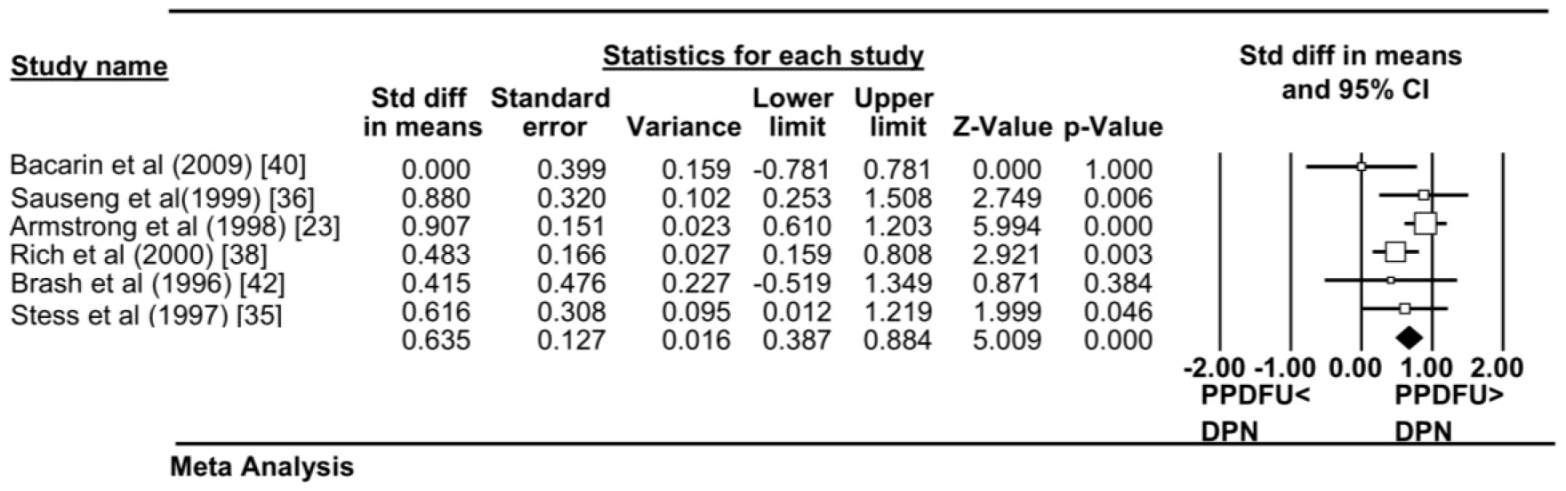
Forest Plot. Forest Plot displaying the Fore Foot Peak Plantar Pressure (MPP) between PPDFU group (cases) and DPN group (control). Overall effect is represented by the coloured diagonal. Six studies are included in total.

As only two studies reported on MPP at the rear foot, meta-analysis was not possible [38], [40]. This was also true with assessment made at the mid foot [40], [42]. However, six studies reported MPP measures at the fore foot (Table S2 in File S1). Meta-analysis combining data from all six studies (PPDFU n = 211; DPN with no foot ulcer history n = 339) suggested that patients with PPDFU had greater fore foot MPP with moderate effect levels (standardised mean difference 0.635, 95% CI 0.387–0.884; p<0.001) (see Figure 4). The heterogeneity between studies was moderate I^2^ = 31.4. When excluding the study by Rich et al. due to differences in unit of analysis, the heterogeneity dropped to I^2^ = 26.7, with a slight increase in the overall effect (Table 2). The exclusion of the study by Stess et al. resulted in a small reduction in effect size and an increase in heterogeneity I^2^ = 44.9. The exclusion of the studies by both Rich et al. and Stess et al. resulted in an increase in effect size but also an increase in heterogeneity levels I^2^ = 42.5 (see Table 2).

#### PTI at various plantar foot regions

Meta-analysis was only possible for fore foot PTI, as there were insufficient studies for comparison of rear foot and mid foot PTI. Meta-analysis combining data from three studies (PPDFU n = 79; DPN with no foot ulcer history n = 54) revealed higher fore foot PTI in PPDFU patients (standardised mean difference 0.719, 95% CI 0.197–1.242; p = 0.007). The heterogeneity between studies was high I^2^ = 44.4 (see Table 2).

### Potential factors affecting plantar pressure measurements in studies

We examined the effect of a number of potential confounding factors including BMI, age and duration of DM on plantar pressure at the aggregate level. Scatterplots of these variables were constructed (Figure S2a, S2b, S2c).These analyses suggested that higher BMI was associated with higher MPP in both groups, which was consistent with previous studies [42], [43]. It was not possible to adjust our analyses for BMI since individual data was not available. Therefore all inferences from aggregate data included in this analysis should be made cautiously.

## Discussion

One of the most detrimental complications of both Type 1 and Type 2 DM is neuropathic foot ulceration [44]. As a majority of neuropathic foot ulcers are likely to form due to mechanical loading on the insensate foot during locomotion, plantar pressure is an important biomechanical consideration in the investigation of the neuropathic foot [14], [45], [46], [47]. This meta-analysis identified that overall MPP was higher in DPN patients with a history of foot ulceration compared to those without a history of foot ulceration, although there was moderate between study heterogeneity. Some patients included in this meta-analysis had active foot ulcers while others had a past history of foot ulceration at the time of assessment. Subgroup analyses revealed only those with previous ulceration, and not active ulceration, had significantly greater plantar pressures compared to those with DPN alone, with low between study heterogeneity. The sample sizes for the sub-group analyses were however small. PTI was significantly higher in DPN patients with a history of foot ulceration. Heterogeneity between studies for this finding was moderate. Sensitivity analyses suggested that heterogeneity between studies assessing MPP was contributed to by a number of factors including study quality and reporting.

Secondary outcome data suggested that patients with PPDFU had higher plantar pressures at the fore foot with moderate between study heterogeneity. Further analysis involving those with previous ulceration only and in those with active ulceration only was not possible. Subgroup analysis was limited by the demographic information available in studies and the overall number of studies. Hence this could not be carried out separately for Type 1 and Type 2 DM, age, BMI, or DM duration. A sensitivity analysis suggested that heterogeneity was reduced by excluding studies in which the SD had to be estimated [33], [34]. One study included patients with minor pedal amputations which have been reported to increase plantar pressures and may have confounded their assessments [48]. Therefore future plantar pressure studies are recommended to adjust for the role of amputation or to include those with a previous minor amputation as a standalone group to determine plantar pressures in this end-stage DPN group. Regardless of these limitation, the finding of elevated fore foot plantar pressures in patients with PPDFU are consistent with the fact that the majority of DFU are found in the forefoot region [49]. PTI assessed at the forefoot was also greater in patients with PPPDFU. Sensitivity and sub-group analyses were unable to be performed due to the low number of available studies. Hence further studies are needed to assess the plantar pressure characteristics of the different anatomical areas of the foot in active and previous foot ulcer populations.

The MPP of patients with active DFUs was not significantly higher than controls; yet, the MPP of patients with previous DFUs was significantly higher. The between study heterogeneity of factors such as different lengths of time since ulceration and different characteristics of DFUs included in the individual studies may have contributed to this result. Small sample sizes were also present which could have contributed to the non-significant result in the active DFU group. Bacarin et al. included only patients who had a previous foot ulcer, and no active ulceration, within the 12 months preceding their examination and this was one of the few studies which reported no difference in plantar pressures between the two groups [40]. However, Sauseng and colleagues utilised active ulcer patients who had a history of at least one ulcer on the contralateral foot [36]. Armstrong et al. only included patients who had an existing or recently healed (<4 weeks) plantar foot ulcer, however the relative number of patients in each category were not defined and this study was unable to be included in subgroup analyses [23]. Boulton et al. included participants with a history of plantar ulcers with neuropathy and painful bilateral symptoms (paraesthesia, burning, and cramps) and absent ankle jerks for at least twelve months preceding the study [33]. For Rich et al. we were unable to distinguish demographic characteristics of the PPDFU and DPN groups from the reported aggregate data and the authors were unable to provide this information when requested [38]. Brash et al. studied feet which were ulcerated on the plantar surface of the first metatarsal head up to 2 years prior to the study [41]. Stess et al. included 67% of patients with active ulcers and 26% of those with active ulcers had some form of lower extremity digital or ray amputation [35]. Ideally, a more homogenous group of DFU participants are needed to confirm plantar pressure changes which accompany previous and active ulceration in those with DPN. Adopting international standard methods for the non-invasive diagnoses of sensory neuropathy may increase homogeneity of future studies and could have improved the homogeneity of this meta-analysis. For example, as identified in our study, there are currently a range of non-invasive methods for diagnosing DPN; however, a more anatomically global tool such as a pressure specific sensory device (PSSD) may provide a more standardised method for diagnosing sensory neuropathy [50]. PSSD has been validated as a non-invasive method to detect sensory neuropathy in a variety of diverse anatomical locations including the foot [50], [51], breast [52] and tongue [53]. Recommendations to standardise non-invasive methods that apply to a broad range of anatomical locations may assist homogeneity of a wide variety of studies in the future.

Although heterogeneity is evident between studies, the results of this meta-analysis seems to suggest that those with DPN and previous ulceration demonstrate an elevation in plantar pressure compared to those with DPN and no ulceration history. However, those with DPN and active ulceration do not demonstrate an elevation in plantar pressures compared to those with DPN and no ulceration history. It could be hypothesised that even though those with active foot ulcers are insensate, they may still alter their movement characteristics to a ‘guarded gait strategy’ during barefoot gait to compensate for the presence of their active ulcer, which in turn may result in a reduction in plantar pressures during the active ulcer phase. This highlights the possibility of an alteration in the gait strategy of these individuals during active ulceration which is contrary to previous findings in the area [12]–[14]. Furthermore, it could be postulated that following ulcer healing, it is likely that this guarded gait strategy diminishes over time and plantar pressures return to the high levels that may have either initiated the active ulcer or were experienced during the phase of any previously healed ulcer. This hypothesis would support studies indicating that identifying a plantar pressure cut-off value to predict ulceration is plausible. Although this theory is consistent with the meta-analysis findings, the current evidence is insufficient to substantiate this hypothesis.

Despite limitations, this meta-analysis supplements the body of research in the area of plantar pressure in patients with DPN and offers evidence for differences in plantar pressure between those with DPN and a history of DFU compared to those with DPN without an ulcer history. This meta-analysis has a number of key limitations including the significant between study heterogeneity, small sample sizes and the lack of available studies. It remains unclear whether screening patients for elevated plantar pressures improves patient outcomes.

## Conclusion

Whilst the potential feasibility of using plantar pressure as a screening tool for ulceration remains viable, more explicit studies, including longitudinal studies involving better defined patient groups are needed to clarify the extent of plantar pressure difference throughout the sequelae of peripheral diabetic complications. Furthermore, while this meta-analysis occurred at an aggregate level, more prospective studies are needed to investigate the role of factors such as BMI, age and DM duration which could potentially influence plantar pressures in those with a history of foot ulcers and active foot ulcers. The use of plantar pressure as a screening tool could be supplemented by further understanding the influence that plantar pressures have in foot ulcer pathogenesis and healing.

## Supporting Information

Figure S1 **Search terms.** Search Terms utilised for database searching.
(TIF)

Figure S2 **Scatterplots of associated variables affecting plantar pressure.** The scatterplots display potential clinical variables influencing the differences in plantar pressure between PPDFU and DPN, at the aggregate level. These included [a] diabetes duration (years), [b] body mass index (BMI) and [c] chronological age.
(TIF)

Checklist S1 **PRISMA guidelines checklist.**
(PDF)

Checklist S2 **MOOSE guidelines checklist.**
(DOCX)

File S1 **Contains two tables Table S1 (Assessment of methodological quality of studies) and Table S2 (Plantar pressure distribution).** Table S1. Assessment of methodological quality of studies. Methodological quality of studies as assessed independently by (EP) and (PL) using a modified quality assessment tool. For the scoring system, 1 =  (P) partially, 0 =  (N) no and 2 =  (Y) yes. The Total score was out of a possible 50. Mean scores were the average of the two individual scores rounded down to the nearest integer. The mean scores in (brackets) indicate mean scores for the assessment of participant and plantar pressure related methodology out of a total of 22 (Q16-Q25). *PVD =  Peripheral Vascular Disease. a These were the subject relevant questions (regarding participant specific characteristics and methods of plantar pressure measurement) which were assessed as suitable by a panel of experts and were added to the quality assessment tool; The identification and quantification of DPN-How was neuropathy diagnosed and quantified? The identification or exclusion of PVD in participants- Was PVD accounted for appropriately? The identification of type of diabetes and diabetes duration in participants- These are important considerations in diabetes foot ulcer pathogenesis. Whether the glycaemic control of the participants' was reported- This gives guidance as to the level of glycemic control of participants. Whether the foot structure of participants was reported- An important consideration in the assessment of plantar pressure. Whether a history of diabetes foot ulceration or current diabetes foot ulceration was checked in all participants? Whether the methods pertaining to plantar pressure capture were reported; this included the general methods, number of steps, verbal instructions and number of walking trials- This was to identify the feasibility for reproducibility of the study using appropriate methods. **Table S2: Plantar pressure distribution.** Reported foot plantar pressures and pressure time integrals normalised to body weight (mean) and (SD). Where multiple results were reported, the highest value in the PPDFU group was used with the corresponding value in the control group. For overall peak pressure, the highest reported MPP and PTI was used, irrespective of location. ^a^This study did not report S D therefore the S D were estimated, the values in brackets indicate estimated S D (please see manuscript for details of how these were approximated). ^b^This study did not report Mean and s.d and in place (IQR) was reported. ^c^This study reported findings as number of feet instead of patients. ^d^This study reported absolute values for MPP but not for PTI, therefore the PTI values were estimated from graphs provided.
(DOCX)

## References

[1] Jeffcoate WJ, Harding KG (2003) Diabetic foot ulcers. The Lancet 361: 1545–1551. 10.1016/S0140-6736(03)13169-8 12737879

[2] Boulton AJ, Vileikyte L, Ragnarson-Tennvall G, Apelqvist J (2005) The global burden of diabetic foot disease. Lancet 366: 1719–1724. 16291066 10.1016/S0140-6736(05)67698-2

[3] Singh N, Armstrong DG, Lipsky BA (2005) Preventing foot ulcers in patients with diabetes. Journal of the American Medical Association 293: 217–228. 15644549 10.1001/jama.293.2.217

[4] Gonzalez R, Pedro T, Real JT, Martinez-Hervas S, Abellan MR, et al (2010) Plasma homocysteine levels are associated with ulceration of the foot in patients with type 2 diabetes mellitus. Diabetes/metabolism research and reviews 26: 115–120. 20135633 10.1002/dmrr.1061

[5] Clayton WJ, Elasy TA (2009) A Review of the Pathophysiology, Classification, and Treatment of Foot Ulcers in Diabetic Patients. Journal of Clinical Diabetes 27: 52–58.

[6] Shenoy AM (2012) Guidelines in practice: Treatment of painful diabetic neuropathy. Continuum Lifelong Learning in Neurology 18: 192–198. 22810079 10.1212/01.CON.0000411562.03591.74

[7] Cook J, Simonson D (2012) Epidemiology and Health Care Cost of Diabetic Foot Problems. In: Veves A, Giurini JM, LoGerfo FW, editors. The Diabetic Foot.Online: Springer. pp.17–32. Available: http://link.springer.com/book/10.1007%2F978-1-61779-791-0. Accessed 18/10/2013

[8] Boulton AJ (2004) The diabetic foot: From art to science. The 18th Camillo Golgi lecture. Diabetologia 47: 1343–1353. 15309286 10.1007/s00125-004-1463-y

[9] Lazzarini PA, Gurr JM, Rogers JR, Schox A, Bergin SM (2012) Diabetes foot disease: the Cinderella of Australian diabetes management? J Foot Ankle Res 5: 24. 23021818 10.1186/1757-1146-5-24 PMC3488529

[10] Cox PS, Williams SK, Weaver SR (2011) Life after lower extremity amputation in diabetics. The West Indian Medical Journal 60: 536–540. 22519229

[11] Garcia-Morales E, Lazaro-Martinez JL, Martinez-Hernandez D, Aragon-Sanchez J, Beneit-Montesinos JV, et al (2011) Impact of diabetic foot related complications on the Health Related Quality of Life (HRQol) of patients-a regional study in Spain. The international Journal of Lower Extremity Wounds 10: 6–11. 21444605 10.1177/1534734611400257

[12] Savelberg HHCM, Schaper NC, Meijer K (2009) The vertical component of the ground reaction force does not reflect horizontal braking or acceleration per se. Clinical Biomechanics 24: 527–528. 19477057 10.1016/j.clinbiomech.2009.05.001

[13] Cavanagh P, Ulbrecht J, Caputo G (1996) Biomechanical aspects of diabetic foot disease: aetiology, treatment, and prevention. Diabet Med 13: S17–22. 8741823

[14] Veves A, Murray HJ, Young MJ, Boulton AJ (1992) The risk of foot ulceration in diabetic patients with high foot pressure: A prospective study. Diabetologia 35: 660–663. 1644245 10.1007/BF00400259

[15] Melai T, Ijzerman TH, Schaper NC, de Lange TLH, Willems PJB, et al (2011) Calculation of plantar pressure time integral, an alternative approach. Gait and Posture 34: 379–383. 21737281 10.1016/j.gaitpost.2011.06.005

[16] Fernando M, Crowther R, Lazzarini P, Sangla K, Cunningham M, et al (2013) Biomechanical characteristics of peripheral diabetic neuropathy: A systematic review and meta-analysis of findings from the gait cycle, muscle activity and dynamic barefoot plantar pressure. Clinical Biomechanics 28: 831–45. 24035444 10.1016/j.clinbiomech.2013.08.004

[17] Bus SA, Waaijman R, Arts M, Manning H (2009) The efficacy of a removable vacuum-cushioned cast replacement system in reducing plantar forefoot pressures in diabetic patients. Clinical Biomechanics 24: 459–464. 19303180 10.1016/j.clinbiomech.2009.02.004

[18] Viswanathan V, Madhavan S, Gnanasundaram S, Gopalakrishna G, Nath Das B, et al (2004) Effectiveness of Different Types of Footwear Insoles for the Diabetic Neuropathic Foot: A follow-up study. Diabetes care 27: 474–477. 14747231 10.2337/diacare.27.2.474

[19] van Deursen R (2008) Footwear for the neuropathic patient: Offloading and stability. Diabetes/metabolism research and reviews 24: S96–S100. 18357582 10.1002/dmrr.827

[20] Paton J, Bruce G, Jones R, Stenhouse E (2011) Effectiveness of insoles used for the prevention of ulceration in the neuropathic diabetic foot: a systematic review. J Diabetes Complications 25: 52–62. 19854075 10.1016/j.jdiacomp.2009.09.002

[21] Coles S (2008) Footwear and offloading for patients with diabetes. Nurs Times 104: : 40, 42–43. 18293878

[22] Lavery LA, Armstrong DG, Vela SA, Quebedeaux TL, Fleischli JG (1998) Practical criteria for screening patients at high risk for diabetic foot ulceration. Arch Intern Med 158: 157–162. 9448554 10.1001/archinte.158.2.157

[23] Armstrong D, Peters E, Athanasiou K, Lavery L (1998) Is there a critical level of plantar foot pressure to identify patients at risk for neuropathic foot ulceration? J Foot Ankle Surg 37: 303–307. 9710782 10.1016/s1067-2516(98)80066-5

[24] Lavery LA, Armstrong DG, Wunderlich RP, Tredwell J, Boulton AJ (2003) Predictive value of foot pressure assessment as part of a population-based diabetes disease management program. Diabetes care 26: 1069–1073. 12663575 10.2337/diacare.26.4.1069

[25] Crawford F, Inkster M, Kleijnen J, Fahey T (2007) Predicting foot ulcers in patients with diabetes: A systematic review and meta-analysis. QJM 100: 65–86. 17277315 10.1093/qjmed/hcl140

[26] Chevalier TL, Hodgins H, Chockalingam N (2010) Plantar pressure measurements using an in-shoe system and a pressure platform: A comparison. Gait & Posture 31: 397–399. 20044257 10.1016/j.gaitpost.2009.11.016

[27] Sanderson S, Tatt ID, Higgins JP (2007) Tools for assessing quality and susceptibility to bias in observational studies in epidemiology: a systematic review and annotated bibliography. Int J Epidemiol 36: 666–676. 17470488 10.1093/ije/dym018

[28] Critical Appraisal Skills Programme C (2010) Critical Appraisal Skills Programme: making sense of evidence about clinical effectiveness: 11 questions to help you make sense of cohort study. Available: http://wwwcasp-uknet/wp-content/uploads/2011/11/CASP-Cohort-Study-Checklist-310513pdf. Accessed: 14th November 2013.

[29] Critical Appraisal Skills Programme C (2010) Critical Appraisal Skills Programme: making sense of evidence about clinical effectiveness: 11 questions to help you make sense of case control study. Available: http://wwwcasp-uknet/wp-content/uploads/2011/11/CASP-Case-Control-Study-Checklist-310513pdf. Accessed: 10th November 2013.

[30] Maher CG, Sherrington C, Herbert RD, Moseley AM, Elkins M (2003) Reliability of the PEDro scale for rating quality of randomized controlled trials. Physical therapy 83: 713–721. 12882612

[31] Stroup DF, Berlin JA, Morton SC (2000) Meta-analysis of observational studies in epidemiology: A proposal for reporting. JAMA: the journal of the American Medical Association 283: 2008–2012. 10789670 10.1001/jama.283.15.2008

[32] Cohen J (1988) Statistical Power analysis of the behavioural sciences. United States: Lawrence Erlbaum Associates Inc. 474 p.

[33] Boulton AJ, Hardisty CA, Betts RP, Franks CI, Worth RC, et al (1983) Dynamic foot pressure and other studies as diagnostic and management aids in diabetic neuropathy. Diabetes Care 6: 26–33. 6839919 10.2337/diacare.6.1.26

[34] Cavanagh PR, Sims DS, Sanders LJ (1991) Body mass is a poor predictor of peak plantar pressure in diabetic men. Diabetes care 14: 750–755. 1954813 10.2337/diacare.14.8.750

[35] Stess RM, Jensen SR, Mirmiran R (1997) The role of dynamic plantar pressures in diabetic foot ulcers. Diabetes care 20: 855–858. 9135955 10.2337/diacare.20.5.855

[36] Sauseng S, Kästenbauer T, Sokol G, Irsigler K (1999) Estimation of risk for plantar foot ulceration in diabetic patients with neuropathy. Diabetes, Nutrition and Metabolism - Clinical and Experimental 12: 189–193. 10554901

[37] McGough JJ, Faraone SV (2009) Estimating the size of treatment effects: moving beyond p values. Psychiatry (Edgmont (Pa: Township)) 6: 21–29. PMC2791668 20011465

[38] Rich J, Veves A (2000) Forefoot and Rearfoot Plantar Pressures in Diabetic Patients: Correlation to Foot Ulceration. Wounds 12: 82–87.

[39] Persaud R (1996) Misleading meta-analysis. "Fail safe N" is a useful mathematical measure of the stability of results. BMJ 312: 125. 10.1136/bmj.312.7023.125 PMC2349768 8555918

[40] Bacarin TA, Sacco IC, Hennig EM (2009) Plantar pressure distribution patterns during gait in diabetic neuropathy patients with a history of foot ulcers. Clinics (Sao Paulo, Brazil) 64: 113–120. 10.1590/S1807-59322009000200008 PMC2666475 19219316

[41] Brash PD, Foster JE, Vennart W, Daw J, Tooke JE (1996) Magnetic resonance imaging reveals micro-haemorrhage in the feet of diabetic patients with a history of ulceration. Diabet Med 13: 973–978. 8946156 10.1002/(SICI)1096-9136(199611)13:11<973::AID-DIA272>3.0.CO;2-5

[42] Shen J, Liu F, Zeng H, Wang J, Zhao JG, et al (2012) Vibrating perception threshold and body mass index are associated with abnormal foot plantar pressure in type 2 diabetes outpatients. Diabetes Technol Ther 14: 1053–1059. 22934798 10.1089/dia.2012.0146 PMC3482851

[43] Sugimoto K, Yasujima M, Yagihashi S (2008) Role of advanced glycation end products in diabetic neuropathy. Curr Pharm Des 14: 953–961. 18473845 10.2174/138161208784139774

[44] Boulton AJ (2005) Management of Diabetic Peripheral Neuropathy. Clinical Diabetes 23: 9–15.

[45] Masson EA, Hay EM, Stockley I, Veves A, Betts RP, et al (1989) Abnormal foot pressures alone may not cause ulceration. Diabetic Medicine 6: 426–428. 2527680 10.1111/j.1464-5491.1989.tb01198.x

[46] Cavanagh P, Ulbrecht J, Caputo G (2000) New developments in the biomechanics of the diabetic foot. Diabetes/metabolism research and reviews 16: S6–S10. 11054880 10.1002/1520-7560(200009/10)16:1+<::aid-dmrr130>3.0.co;2-z

[47] van Schie CH (2005) A review of the biomechanics of the diabetic foot. The international journal of lower extremity wounds 4: 160–170. 16100097 10.1177/1534734605280587

[48] Armstrong DG, Lavery LA (1998) Plantar pressures are higher in diabetic patients following partial foot amputation. Ostomy Wound Manage 44: 30–32. 9626005

[49] Altindas M, Kilic A, Cinar C, Bingol UA, Ozturk G (2011) The Epidemiology of Foot Wounds in Patients with Diabetes: A Description of 600 Consecutive Patients in Turkey. The Journal of Foot & Ankle Surgery 50: 146–152. 21353997 10.1053/j.jfas.2010.12.017

[50] Wood WA, Wood MA, Werter SA, Menn JJ, Hamilton SA, et al (2005) Testing for loss of protective sensation in patients with foot ulceration: a cross-sectional study. J Am Podiatr Med Assoc 95: 469–474. 16166466 10.7547/0950469

[51] Ferreira MC, Rodrigues L, Fels K (2004) New method for evaluation of cutaneous sensibility in diabetic feet: preliminary report. Rev Hosp Clin Fac Med Sao Paulo 59: 286–290. 15543401 10.1590/s0041-87812004000500011

[52] Longo B, Campanale A, Farcomeni A, Santanelli F (2013) Long-term sensory recovery of nipple-areola complex following superolateral pedicled reduction mammaplasty. Plast Reconstr Surg 132: 735–742. 10.1097/PRS.0b013e3182a3bf05 24165625

[53] Longo B, Pagnoni M, Ferri G, Morello R, Santanelli F (2013) The mushroom-shaped anterolateral thigh perforator flap for subtotal tongue reconstruction. Plast Reconstr Surg 132: 656–665. 23985639 10.1097/PRS.0b013e31829acf84

