# Supplementary figures and images for: Plantar Pressure in Diabetic Peripheral Neuropathy Patients with Active Foot Ulceration, Previous Ulceration and No History of Ulceration: A Meta-Analysis of Observational Studies
Source: PLoS One. 2014 Jun 10;9(6):e99050. doi: 10.1371/journal.pone.0099050 (PMC4051689; doi:10.1371/journal.pone.0099050)

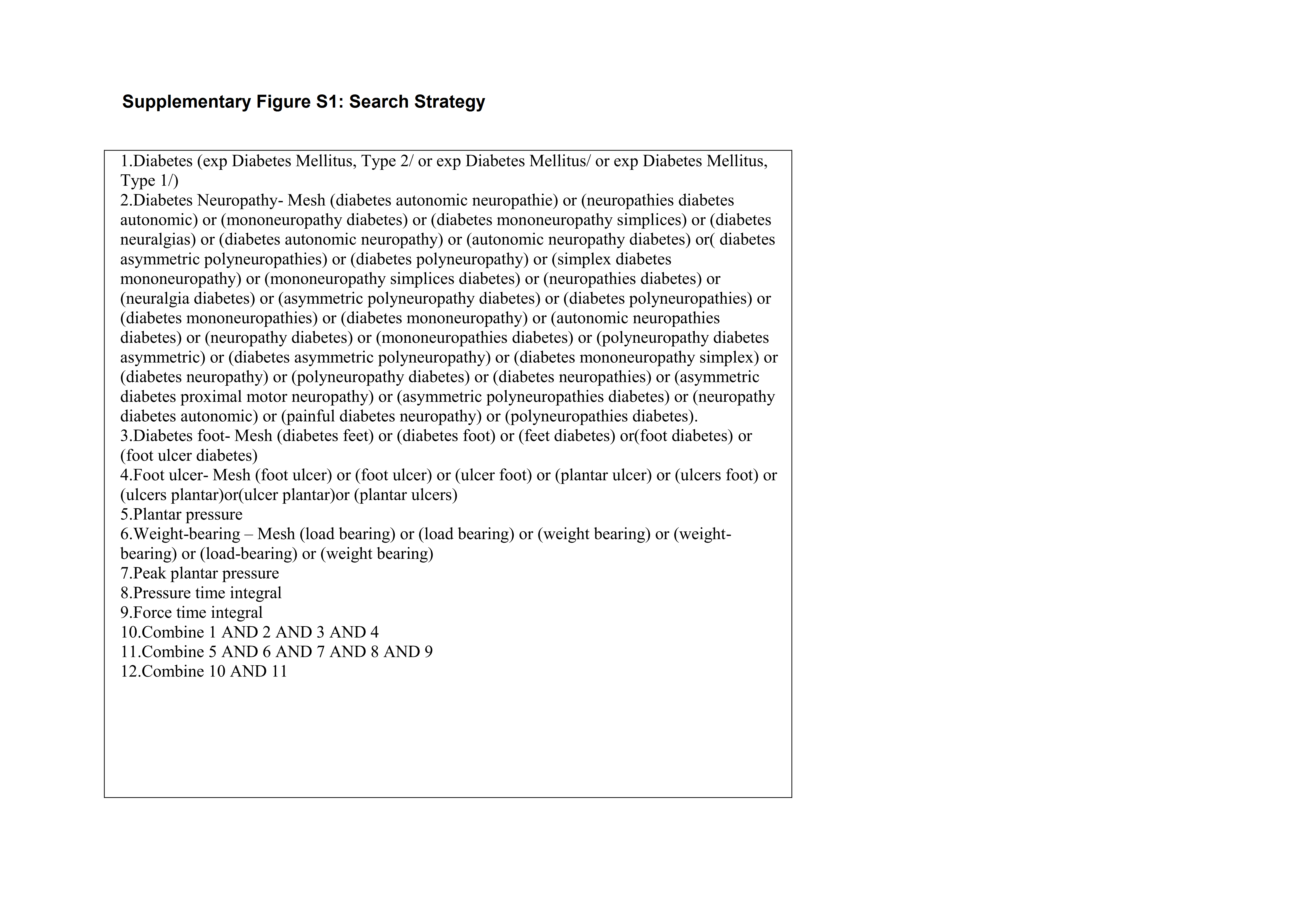

Supplement: Figure S1 — Search terms. Search Terms utilised for database searching. (TIF) [file pone.0099050.s001.tif]

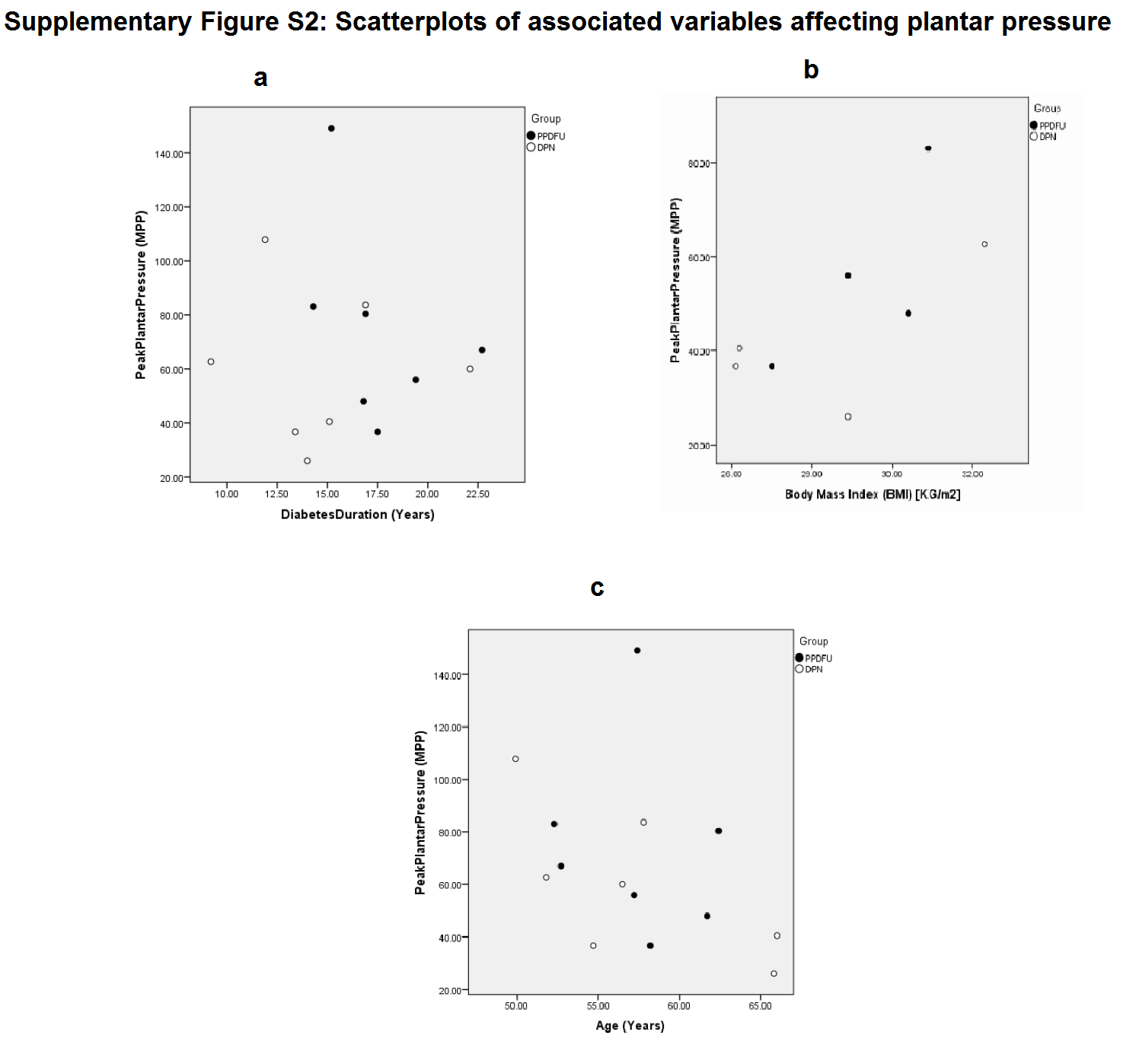

Supplement: Figure S2 — Scatterplots of associated variables affecting plantar pressure. The scatterplots display potential clinical variables influencing the differences in plantar pressure between PPDFU and DPN, at the aggregate level. These included [a] diabetes duration (years), [b] body mass index (BMI) and [c] chronological age. (TIF) [file pone.0099050.s002.tif]
