# Supplementary material for: Plantar Pressure in Diabetic Peripheral Neuropathy Patients with Active Foot Ulceration, Previous Ulceration and No History of Ulceration: A Meta-Analysis of Observational Studies
Source: PLoS One. 2014 Jun 10;9(6):e99050. doi: 10.1371/journal.pone.0099050 (PMC4051689; doi:10.1371/journal.pone.0099050)
Supplement: Checklist S2 — MOOSE guidelines checklist. (DOCX) [file pone.0099050.s004.docx]

**MOOSE Checklist**

From: Stroup DF, Berlin JA, Morton SC, et al (2000) Meta-analysis of observational studies in epidemiology: A proposal for reporting. JAMA 283:2008–2012. doi:10.1001/jama.283.15.2008.

|  | Reported on page | Comments |
| --- | --- | --- |
| **Reporting of background should include** | | |
| Problem definition | Introduction |  |
| Hypothesis statement | Introduction |  |
| Description of study outcome(s) | Methods |  |
| Type of exposure or intervention used |  | Does not apply |
| Type of study designs used | Methods |  |
| Study population | Introduction/Methods |  |
| **Reporting of search strategy should include** | | |
| Qualifications of searchers (e.g. librarians and investigators) | Search strategy |  |
| Search strategy, including time period used in the synthesis and key words | Search strategy |  |
| Effort to include all available studies, including contact with authors | Study selection |  |
| Databases and registries searched | Supplementary Figure 1 |  |
| Search software used, name and version, including special features used (e.g. explosion) | Search Strategy |  |
| Use of hand searching (e.g. reference lists of obtained articles) | Search Strategy |  |
| List of citations located and those excluded, including justification | Supplementary Figure 1 |  |
| Method of addressing articles published in languages other than English | Data extraction and synthesis |  |
| Method of handling abstracts and unpublished studies | Data extraction and synthesis |  |
| Description of any contact with authors | Data extraction and synthesis |  |
| **Reporting of methods should include** | | |
| Description of relevance or appropriateness of studies assembled for assessing the hypothesis to be tested | Search Results |  |
| Rationale for the selection and coding of data (e.g. sound clinical principles or convenience) |  | Does not apply |
| Documentation of how data were classified and coded (e.g. multiple raters, blinding and interrater reliability) | Risk of Bias and Data extraction and synthesis |  |
| Assessment of confounding (e.g. comparability of cases and controls in studies where appropriate) |  | Does not apply |
| Assessment of study quality, including blinding of quality assessors, stratification or regression on possible predictors of study results | Risk of Bias |  |
| Assessment of heterogeneity | Risk of Bias |  |
| Description of statistical methods (e.g. complete description of fixed or random effects models, justification of whether the chosen models account for predictors of study results, dose-response models, or cumulative meta-analysis) in sufficient detail to be replicated | Statistical methods |  |
| Provision of appropriate tables and graphics | See figures and tables |  |
| **Reporting of results should include** | | |
| Graphic summarizing individual study estimates and overall estimate | See Figures 1-3 |  |
| Table giving descriptive information for each study included | See Table 1 |  |
| Results of sensitivity testing (e.g. subgroup analysis) | See Table 2 |  |
| Indication of statistical uncertainty of findings | Table 2 |  |
| **Reporting of discussion should include** | | |
| Quantitative assessment of bias (e.g. publication bias) | Risk of Bias |  |
| Justification for exclusion (e.g. exclusion of non-English language citations) | Study selection |  |
| Assessment of quality of included studies | Risk of Bias | See Supplementary Table 1 |
| **Reporting of conclusions should include** | | |
| Consideration of alternative explanations for observed results | Discussion |  |
| Generalization of the conclusions (i.e. appropriate for the data presented and within the domain of the literature review) | Discussion |  |
| Guidelines for future research | Discussion |  |
| Disclosure of funding source | Manuscript |  |
