# Supplementary material for: Plantar Pressure in Diabetic Peripheral Neuropathy Patients with Active Foot Ulceration, Previous Ulceration and No History of Ulceration: A Meta-Analysis of Observational Studies
Source: PLoS One. 2014 Jun 10;9(6):e99050. doi: 10.1371/journal.pone.0099050 (PMC4051689; doi:10.1371/journal.pone.0099050)
Supplement: File S1 — Contains two tables Table S1 (Assessment of methodological quality of studies) and Table S2 (Plantar pressure distribution). Table S1. Assessment of methodological quality of studies. Methodological quality of studies as assessed independently by (EP) and (PL) using a modified quality assessment tool. For the scoring system, 1 = (P) partially, 0 = (N) no and 2 = (Y) yes. The Total score was out of a possible 50. Mean scores were the average of the two individual scores rounded down to the nearest integer. The mean scores in (brackets) indicate mean scores for the assessment of participant and plantar pressure related methodology out of a total of 22 (Q16-Q25). *PVD = Peripheral Vascular Disease. a These were the subject relevant questions (regarding participant specific characteristics and methods of plantar pressure measurement) which were assessed as suitable by a panel of experts and were added to the quality assessment tool; The identification and quantification of DPN-How was neuropathy diagnosed and quantified? The identification or exclusion of PVD in participants- Was PVD accounted for appropriately? The identification of type of diabetes and diabetes duration in participants- These are important considerations in diabetes foot ulcer pathogenesis. Whether the glycaemic control of the participants' was reported- This gives guidance as to the level of glycemic control of participants. Whether the foot structure of participants was reported- An important consideration in the assessment of plantar pressure. Whether a history of diabetes foot ulceration or current diabetes foot ulceration was checked in all participants? Whether the methods pertaining to plantar pressure capture were reported; this included the general methods, number of steps, verbal instructions and number of walking trials- This was to identify the feasibility for reproducibility of the study using appropriate methods. Table S2: Plantar pressure distribution. Reported foot plantar pressures an [file pone.0099050.s005.docx]

**Supplementary Table S1: Assessment of methodological quality of studies**

|  | **Study** | **Bacarin et al.**  **2009 [40]** | | **Brash et al.**  **1996 [41]** | | **Armstrong et al. 1998 [23]** | | **Stess et al.**  **1997 [35]** | | **Rich et al.**  **2000 [38]** | | **Sauseng et al.**  **1999 [36]** | | **Cavanagh et al.**  **1991 [34]** | | **Boulton et al.**  **1983 [33]** | | |
| --- | --- | --- | --- | --- | --- | --- | --- | --- | --- | --- | --- | --- | --- | --- | --- | --- | --- | --- |
| **Q No.** | **Question** | **EP** | **PL** | **EP** | **PL** | **EP** | **PL** | **EP** | **PL** | **EP** | **PL** | **EP** | **PL** | **EP** | **PL** | **EP** | **PL** |  |
| 1 | Clear Aim/Purpose | Y | Y | Y | Y | Y | Y | P | Y | P | Y | Y | Y | Y | Y | P | Y |  |
| 2 | Suitable Design was used? | P | P | Y | P | P | P | Y | P | Y | Y | P | P | Y | P | Y | P |  |
| 3 | Efficient recruitment strategy used? | Y | P | Y | P | Y | P | Y | Y | P | P | Y | P | Y | P | Y | P |  |
| 4 | Suitable exclusion and inclusion criteria? | Y | P | Y | P | P | P | P | P | P | P | P | Y | Y | P | Y | P |  |
| 5 | Data reported for at least 85% on a key outcome? | Y | Y | Y | Y | Y | Y | Y | Y | Y | Y | Y | Y | Y | Y | Y | P |  |
| 6 | Discussed main sources of bias? | Y | Y | N | N | Y | P | N | N | N | N | Y | P | Y | Y | N | N |  |
| 7 | Were the methods justified? | Y | Y | P | Y | P | Y | Y | P | Y | Y | N | N | Y | Y | Y | Y |  |
| 8 | Was a power calculation for sample conducted? | N | N | N | N | N | N | N | N | N | N | N | N | N | N | N | N |  |
| 9 | Were Confounders identified? | Y | Y | P | Y | Y | Y | P | P | Y | Y | Y | Y | P | Y | P | Y |  |
| 10 | Confounders accounted for in analysis? | Y | Y | P | P | Y | Y | Y | P | Y | Y | Y | Y | Y | Y | P | Y |  |
| 11 | Between group results reported for at least one key outcome? | Y | Y | Y | Y | Y | Y | Y | Y | Y | Y | Y | Y | Y | Y | Y | P |  |
| 12 | Compared findings to literature? | Y | Y | Y | Y | Y | P | Y | Y | Y | Y | Y | Y | Y | Y | Y | Y |  |
| 13 | Findings relevant to aims? | Y | Y | Y | P | P | P | Y | P | Y | Y | Y | Y | Y | Y | P | P |  |
| 14 | Results statistically appropriate? | Y | Y | N | N | P | Y | P | Y | Y | Y | P | Y | N | N | N | N |  |
| 15 | Are findings applicable to a clinical scenario? | P | Y | Y | P | Y | Y | Y | Y | Y | Y | Y | Y | Y | Y | Y | P |  |
| 16^a^ | Assessed degree of neuropathy of participants? | Y | Y | Y | Y | P | P | P | P | Y | Y | Y | Y | Y | Y | Y | Y |  |
| 17 ^a^ | PVD* identified in participants or excluded? | N | N | Y | Y | Y | Y | Y | Y | Y | Y | Y | Y | N | N | Y | Y |  |
| 18 ^a^ | Diabetes duration and type of diabetes identified? | Y | Y | P | P | Y | P | P | P | P | Y | Y | Y | Y | Y | Y | Y |  |
| 19 ^a^ | Level of glycaemic control of participants was reported (i.e. hbA1c)? | Y | Y | N | N | N | N | N | N | N | N | Y | Y | N | N | N | N |  |
| 20 ^a^ | Foot structure reported? | P | P | N | N | N | N | N | N | P | P | P | P | P | P | N | N |  |
| 21 ^a^ | Absence/ presence of current/ past foot ulceration identified? | Y | Y | Y | Y | Y | Y | Y | Y | Y | P | Y | P | Y | P | Y | Y |  |
| 22 ^a^ | Does the study specify methods of plantar pressure capture and analysis? | P | P | P | P | P | P | P | P | P | P | Y | P | Y | P | P | P |  |
| 23 ^a^ | Does the study state the number of steps used in capture? | Y | Y | Y | Y | Y | Y | P | Y | Y | Y | Y | Y | Y | Y | N | N |  |
| 24 ^a^ | Study specifies any verbal instructions given to participants? | Y | Y | N | N | N | N | N | N | Y | Y | N | N | N | N | N | N |  |
| 25 ^a^ | Number of walking trials reported? | P | Y | N | N | N | N | Y | Y | Y | Y | N | N | P | Y | N | N |  |
| Total | Total independent quality scores | 41 | 41 | 31 | 28 | 33 | 31 | 32 | 31 | 38 | 39 | 38 | 36 | 37 | 34 | 29 | 26 |  |
| Mean | Mean Scores | 41 (15) | | 29 (10) | | 32 (9) | | 31 (10) | | 38 (15) | | 37 (14) | | 35 (11) | | 27 (9) | | |

**Supplementary Table S2: Plantar pressure distribution**

| **Study** | **Rear foot** | | **Mid foot** | | **Fore foot** | | **Overall Plantar Pressure** | |
| --- | --- | --- | --- | --- | --- | --- | --- | --- |
| **Group** | PPDFU | DPN | PPDFU | DPN | PPDFU | DPN | PPDFU | DPN |
| **Bacarin 2009 [40]** | | | | | | | | |
| *MPP (N/cm^2^)* | 34.2 (11.9) | 34.2 (7.6) | 29.0 (15.1) | 20.5 (11.8) | 36.7 (8.6) | 36.7 (8.9) | 36.7 (8.6) | 36.7 (8.9) |
| *PTI (Ns/Cm^2^)* | 10.2 (3.7) | 9.4 (2.9) | 6.8 (3.6) | 4.3 (0.9) | 12.5 (3.3) | 11.9 (3.1) | 12.9 (3.3) | 11.9 (3.1) |
| **Cavanagh^a^ [34]**  **1991** | | | | | | | | |
| *MPP (N/cm^2^)* |  | | | | | | 80.4 (34.2) | 83.7 (35.8) |
| **Sauseng^b^ 1999 [36]** | | | | | | | | |
| *MPP (N/cm^2^)* |  | | 10.5 (11.1) | 20.5 (19.3) | 56.0 (48.2) | 26.0  (12.6) | 56.0 (48.2) | 26.0(12.6) |
| *PTI (Ns/Cm^2^)* |  |  | 3.8 (3.5) | 8.1 (8.0) | 23.2 (16.6) | 9.4  (4.7) | 23.2 (16.6) | 9.4 (4.7) |
| **Armstrong**  **1998 [23]** | | | | | | | | |
| *MPP (N/cm^2^)* |  | | | | 83.1 (24.7) | 62.7 (21.4) | 83.1 (24.7) | 62.7 (21.4) |
| **Boulton [33]**  **1983^a^** | | | | | | | | |
| *MPP (N/cm^2^)* |  | | | | | | 149.06 (67.7) | 107.87 (47.6) |
| **Rich ^c^**  **2000 [38]** | | | | | | | | |
| *MPP (N/cm2)* | 34.0 (18.0) | 31.0 (15.0) |  | | 82.0 (43.0) | 66 (28.0) | 82.0 (43.0) | 66.0 (28.0) |
| **Brash**  **1996 [41]** | | | | | | | | |
| *MPP (N/cm2)* |  | | | | 67.0 (20.0) | 60.0 (13.0) | 67.0 (20.0) | 60.0 (13.0) |
| **Stess**  **1997^a, d^ [35]** | | | | | | | | |
| *MPP (N/cm2)* |  | | | | 48.0 (12.6) | 40.5 (10.5) | 48.0 (12.6) | 40.5 (10.5) |
| *PTI (Ns/Cm2)* |  | | | | 32.0 (14.0) | 23 (7.5) | 32.0 (14.0) | 23 (7.5) |
